# A Novel Theoretical Probabilistic Model for Opportunistic Routing with Applications in Energy Consumption for WSNs

**DOI:** 10.3390/s21238058

**Published:** 2021-12-02

**Authors:** Christian E. Galarza, Jonathan M. Palma, Cecilia F. Morais, Jaime Utria, Leonardo P. Carvalho, Daniel Bustos, Ricardo C. L. F. Oliveira

**Affiliations:** 1 Escuela Superior Politécnica del Litoral—ESPOL, Facultad de Ciencias Naturales y Matemáticas, Vía Perimetral 5, Guayaquil 090150, Ecuador; chedgala@espol.edu.ec; 2 Department of Electrical Engineering, Faculty of Engineering, University of Talca, Curicó 3344158, Chile; 3 Center of Exact, Environmental and Technological Sciences, Pontifical Catholic University of Campinas, Campinas 13086-900, SP, Brazil; cecilia.morais@puc-campinas.edu.br; 4 Institute of Mathematics and Statistics, Fluminense Federal University—UFF, Niterói 24210-201, RJ, Brazil; jutria@id.uff.br; 5 Discrete Technology and Production Automation (DTPA), Rijksuniversiteit Groningen, 9712 CP Groningen, The Netherlands; 6 Polytechnic School, University of São Paulo, São Paulo 05508-900, SP, Brazil; carvalho.lp@usp.br; 7 Centro de Investigación de Estudios Avanzados del Maule (CIEAM), Vicerrectoría de Investigación y Postgrado, Universidad Católica del Maule, Talca 3460000, Chile; 8 Laboratorio de Bioinformática y Química Computacional (LBQC), Facultad de Medicina, Universidad Católica del Maule, Talca 3460000, Chile; dbustos@ucm.cl; 9 Escuela de Bioingeniería Médica, Facultad de Medicina, Universidad Católica del Maule, Talca 3460000, Chile; 10 School of Electrical and Computer Engineering, University of Campinas—UNICAMP, Campinas 05508-900, SP, Brazil; ricfow@dt.fee.unicamp.br

**Keywords:** multi-hop network, semi-reliable communication network, opportunistic routing network

## Abstract

This paper proposes a new theoretical stochastic model based on an abstraction of the opportunistic model for opportunistic networks. The model is capable of systematically computing the network parameters, such as the number of possible routes, the probability of successful transmission, the expected number of broadcast transmissions, and the expected number of receptions. The usual theoretical stochastic model explored in the methodologies available in the literature is based on Markov chains, and the main novelty of this paper is the employment of a percolation stochastic model, whose main benefit is to obtain the network parameters directly. Additionally, the proposed approach is capable to deal with values of probability specified by bounded intervals or by a density function. The model is validated via Monte Carlo simulations, and a computational toolbox (R-packet) is provided to make the reproduction of the results presented in the paper easier. The technique is illustrated through a numerical example where the proposed model is applied to compute the energy consumption when transmitting a packet via an opportunistic network.

## 1. Introduction

In wireless ad hoc networks, the traditional cellular architecture for multi-hop routing is characterized by the use of communication schemes where the single objective is the transmission between nodes, e.g., hop-by-hop and end-to-end [1,2]. However, such schemes lack an important property inherent to broadcast wireless channels: simultaneous reception of a single message in multiple nodes. In this case, a transmitted packet can reach more than one unit in a neighborhood (multi-cast coverage) around some destinations (sink). A communication scheme where the transmitted messages can be received by several units corresponds to an Opportunistic Routing (OR) network (ExOR wireless network protocol), formulated in [3,4,5]. This particular structure aims to obtain a higher transmission success rate, lower average delay and even smaller energy consumption when compared with traditional transport strategies [6,7,8,9,10,11]. Those advantages can be achieved because, when the opportunistic routing scheme is implemented, each hop moves the packet farther (on average) than the hops of the best possible predetermined route, implying that the packets may reach the sink using a lower number of hops or jumps [3,12].

Issues such as reduction of the number of transmissions, congestion, and coordination of duplicated messages in ad hoc networks employing OR have been extensively investigated in the literature [10,12,13,14,15]. Many research efforts in this area have been performed by means of network simulators [16,17], i.e., using software like Unicast and Multicast Network Simulator, version 2 or 3 (ns-2 and ns-3). On the other hand, in [3,4,8,10,13], one can find some mathematical formulas for the computation of the success probability, the number of transmissions and the cost functions for selecting the best jump. However, to the best of the authors’ knowledge, there are not theoretical models (i.e., without appealing to approximations via simulation) to compute energy consumption in Wireless Sensor Networks (WSN).

The digital network modeling is certainly a challenging task because phenomena such as error channel (physical layer) and congestion, collision, and error in the data packetization (Data Link and transport layer) can be complex to model in terms of analytical equations, being commonly used support algorithms for the equations [18,19,20,21,22]. However, as previously mentioned, the simulation and emulation of networks in the laboratory can be an extensive and complex task. In this sense, abstracting a mathematical entity that models the flow of packets through closed formulas can be considered an idealized model of the network. The use of theoretical models in the context of transportation or allocation of elements from one point to another in a network corresponds to supply-demand networks (SDN) [23], where, as in WSN, it is of interest to study the probability of a successful delivery, or the computation and probability distribution of the possible routes. In this context, the topology and success probabilities of the routes in the network depend on inherent features of the system, such as distances, intermediate hubs, etc. As noted, problems in SDN are similar to the ones in WSN, where the simulation, even for small systems, may take some time and be costly. For this reason, the use of probabilistic models results as a fast and cost-free solution to these problems, possibly leading to exact results in a split second. The networks parameters can be used as values of references (initial or control values) for future real-life experiments or simulations. Furthermore, these models can be extended to more realistic environments by using uncertain or random coefficients.

This paper proposes a probabilistic model for OR networks capable to compute the law of route formation, probability of successful transmission per route and probability of successful network transmission (source to sink). To the best of the authors’ knowledge, the proposed technique is the first one capable of obtaining these network parameters analytically. Unlike the models available in the literature that use matrix forms based on Markov chains [24,25,26,27], the proposed approach is based on a discrete percolation model, which uses the directed graph theory, favoring the construction of an iterative model. Closed expressions are provided to compute the expected value of the number of broadcasts, packet transmissions and packet receptions. The latter is a useful parameter in network design because it is directly associated with energy consumption cost. The analytical computation of energy consumption (without using a network simulation) minimizes the complexity of the model and provides quick results, making the energy planning of the units easier. Another novelty of the proposed technique is the robustness of the results with respect to the values of the probabilities of transition among modes. To obtain more accurate results, the probabilities can be considered uncertain and structured in two different ways: (i) The probabilities belong to intervals whose upper and lower bounds are known; (ii) the probabilities are random parameters whose probability density function, which depends on the distance between the nodes, is known. Both cases are related to communication channel noise problems. The investigated network presents a linear topology [26] [Chapter 3.4.1], [27,28]. The proposal is validated via Monte Carlo (MC) simulation and a practical example related to energy consumption in WSN is also provided. All methods involved are available for interested readers in the free software (*The R Project for Statistical Computing*—[29]) R through the Package ‘Opportunistic’ [30], providing the full routing scheme, probability of successful transmission, expected number of transmissions, receptions and broadcasts, as well as their respective estimations via MC simulations for a given model.

## 2. Network Definition and Preliminary Discussions

**Figure 1:**
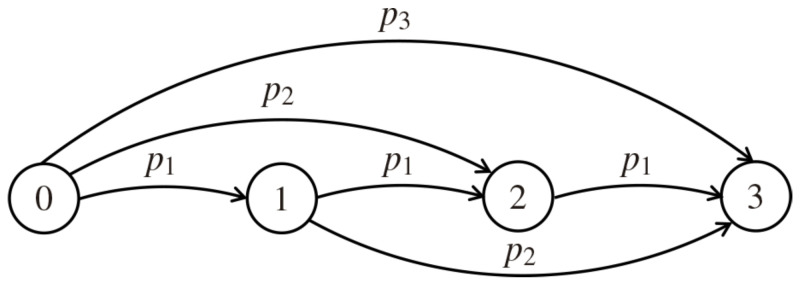
Simple opportunistic network for N=3.

In general, a digital network can be viewed as a collection of nodes located in some space, such that each node can be a transmitter or a receiver. The terminology and the probabilistic model of the end-to-end and hop-by-hop transport schemes for Bernoulli loss process in the channel used in this paper are explained in detail in [31,32]. The network consists of N+1 nodes, labeled 0,1,…,N, where *N* is a finite natural number. At a given instant of time, several nodes transmit simultaneously, each one towards its own receiver. Each transmitter–receiver pair requires its own link. The simplest model of *N*-hop opportunistic network is analyzed, such that the power signal decays with the distance, in a probabilistic way. To this end, for each transmitter node *i* (0≤i≤N−1), it is assigned a vector of probabilities (not necessarily summing one), $p^{\left(i\right)}=\left[p_{1},\ldots ,p_{N-i}\right]$, i=0,…,N−1, such that the *j*th element of $p^{\left(i\right)}$ is the probability of a signal transmitted by node *i* be received by node i+j. Furthermore, it is considered a linear topology network [26,27,28], where nodes (units) are linearly spaced between the source and sink. It is also important to recall that the model does not evolve in time, i.e., all transmission attempts (successful or not) occur simultaneously. Figure 1 shows a simple opportunistic network sample.

One of the issues that must be addressed by the network model is the successful transmission, i.e., when a signal initially transmitted by the source (node 0) reaches the sink (node *N*) at least once (regardless of the traveled route). The OR network model is related to some dependent discrete percolation model [33]. The diagram depicted in Figure 1 can be seen as a directed *multigraph* (DMG), named *directed* because each edge has a direction (from source to sink) and *multigraph* because multiple edges can have the same end nodes. A DMG is denoted by $\vec{F}=\left(V,\vec{E}\right)$, where *V* is a set of nodes and $\vec{E}$ is a multiset of ordered pairs of nodes, called edges. Each directed edge $ij\in \vec{E},i<j,\forall i,j\in V$, is declared open when it has probability $p_{j-i}>0$ and closed otherwise. As noted, the probabilities $p_{j-i}$ depend on the distance j−i between the transmitter node *i* and receiver node *j*. As a consequence, the possible set of probabilities is $\left[p_{1},p_{2},\ldots ,p_{N}\right]$. Many applications of DMG are usually studied in basic probability courses, such as water supply networks, electrical circuits, among others. For instance, suppose that the objective is to supply water from node 0 to *N*. Consider open edges those open to the passage of water. Then, one is naturally interested in answering if the water supplied from node 0, which flows along the open edges only, can reach node *N*. An affirmative answer can be viewed, in fact, as a successful transmission for an opportunistic model.

**Figure 2:**
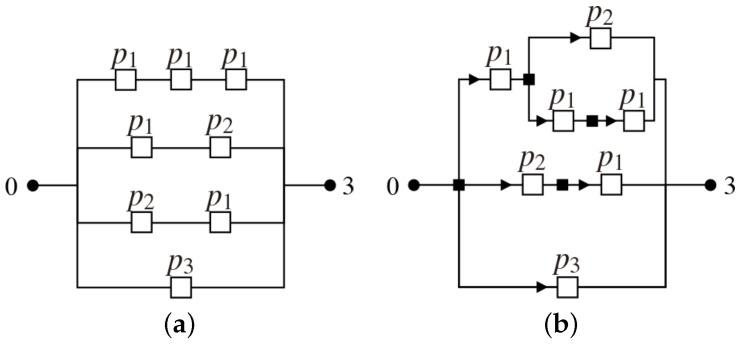
Alternative configurations of the opportunistic network model presented in Figure 1.

Both models depicted in Figure 2 are equivalent to the opportunistic model of 3-hop presented in Figure 1. Squares symbolize the edges that, for the supplying water example, can represent gates, dams or, pumps. Differently from Figure 2a, where the four possible routes from node 0 to node 3 are shown separately, Figure 2b embedded the 3-hop opportunistic network of Figure 2a in 2-hop in the upper route and into a 1-hop in the middle route. This nested scheme leads to recursive formulas presented in the next subsections for the total number of possible routes, the probability of a successful transmission and the expected number of transmissions, receptions, and broadcasts.

### Possible Routes

First, let $K^{N}$ denote the set of the different possible routes in an *N*-hop opportunistic network. This is given by

$$K^{N}=\left\{\kappa =\left(k_{1},\ldots ,k_{N}\right)\in N^{N}:\sum_{i=1}^{N}i\;k_{i}=N\right\}.$$

There exist $\#(K^{N})$ different possible routes in an *N*-hop opportunistic network (in terms of the probability), where #(·) represents the cardinality of a given set. For instance, for the 3-hop system shown in Figure 2, $K^{N}$ contains three solution vectors

$$\kappa_{1}=\left(3,0,0\right),\;\kappa_{2}=\left(1,1,0\right)\;\mathrm{and}\;\kappa_{3}=\left(0,0,1\right),$$

satisfying

$$\sum_{i=1}^{3}ik_{i}=k_{1}+2k_{2}+3k_{3}=3.$$

Each possible route is characterized by a vector κ, whose elements $k_{i}$ represent the number of times that an *i*-hop jump occurs, e.g., $\kappa_{1}=\left(3,0,0\right)$ represents the route (from 0 to 3) as three single jumps, while $\kappa_{3}=\left(0,0,1\right)$ indicates the route as one triple jump. Besides, let $p^{\kappa }$ denote $p_{1}^{k_{1}}p_{2}^{k_{2}}\ldots p_{N}^{k_{N}}$. Thus, one can easily compute the probabilities for each possible route as

$$\begin{array}{cc} p^{\kappa_{1}} & =p_{1}^{3}p_{2}^{0}p_{3}^{0}=p_{1}^{3},\\ p^{\kappa_{2}} & =p_{1}^{1}p_{2}^{1}p_{3}^{0}=p_{1}p_{2},\\ p^{\kappa_{3}} & =p_{1}^{0}p_{2}^{0}p_{3}^{1}=p_{3}. \end{array}$$

Note that the route $\kappa_{2}$ may occur as $p_{1}p_{2}$ or $p_{2}p_{1}$, so one must consider all possible permutations. For this, one can use the multinomial coefficient

$$C\left(\kappa \right)=\frac{(k_{1}+k_{2}+\cdots +k_{N})!}{k_{1}!k_{2}!\ldots k_{N}!},$$

**Figure 3:**
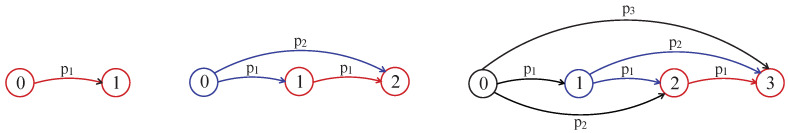
*N*-hop opportunistic models for N=1,2,3. Colors are used to depict that an *N*-hop opportunistic model has N−1 opportunistic submodels embedded.

Then, the three different routes above occur with frequencies

$$\begin{array}{c} C\left(\kappa_{1}\right)=\frac{(3+0+0)!}{3!0!0!}=\frac{3!}{3!}=1,\\ C\left(\kappa_{2}\right)=\frac{(1+1+0)!}{1!1!0!}=\frac{2!}{1!}=2,\\ C\left(\kappa_{3}\right)=\frac{(0+0+1)!}{0!0!1!}=\frac{1!}{1!}=1, \end{array}$$

respectively. Hence, the total number of possible routes for an *N*-hop opportunistic model is given by $N_{R}\left(N\right)=\sum_{\kappa \in K^{N}}C\left(\kappa \right)$. For the previous example, one obtains

$$N_{R}\left(N\right)=C\left(\kappa_{1}\right)+C\left(\kappa_{2}\right)+C\left(\kappa_{3}\right)=1+2+1=4,$$

that is, the four routes that can be easily seen from Figure 3. Furthermore, it is possible to find an analytical expression for $N_{R}\left(N\right)$ by using a recursive approach as presented in what follows.

Figure 3 shows how an *N*-hop opportunistic model can be composed by N−1 opportunistic submodels with number of hops N=1,…,N−1. Thereby, the total number of routes can be summarized as the number of possible routes for all the N−1 submodels, plus the direct route, i.e., from 0 to *N*. This can be written as

(1) $$\begin{array}{cc} N_{R}\left(N\right) & =1+\sum_{i=1}^{N-1}N_{R}\left(i\right). \end{array}$$

From Equation (1), one obtains

$$\begin{array}{cc} N_{R}\left(N+1\right) & =1+\sum_{i=1}^{N}N_{R}\left(i\right)\\ \; & =1+\sum_{i=1}^{N-1}N_{R}\left(i\right)+N_{R}\left(N\right)\\ \; & =2N_{R}\left(N\right), \end{array}$$

leading to the relationship

$$\frac{N_{R}\left(N+1\right)}{N_{R}\left(N\right)}=2,$$

for N≥1. Finally, since $N_{R}\left(1\right)=1$, i.e., there exists only one route in a single hop opportunistic model, one concludes that the total number of routes in an *N*-hop opportunistic model is given by

(2) $$\begin{array}{cc} N_{R}\left(N\right) & =2^{N-1}. \end{array}$$

Different routes with their frequencies and probabilities are provided by the routes() function available in the proposed R package. The results provided by this function in a particular example can be found in the Appendix A section, which corroborates the findings in this section.

## 3. Full Stochastic Opportunist Model

This section presents the probabilistic model that allows the calculation of the probability of successful transmission, expected number of transmissions (assuming independent routes), receptions, and broadcast transmissions. Broadcast is associated with energy costs and is represented in Figure 2b by the squares (▪). Each broadcast generates at least one transmission (▸), such that the expected value of transmissions allows to compute the expected value of reception of broadcasted messages.

### 3.1. Probability of Successful Transmission

Let $P_{S}^{\left(N\right)}$ denote the probability of a successful transmission for an *N*-hop opportunistic model. As an opportunistic network is, in fact, a parallel network, one has

(3) $$P_{S}^{\left(N\right)}=1-\prod_{i=1}^{N}P\left(O_{i}^{c}\right)$$

where $P(O_{i})$ represents the probability that the *i*-th route is open and $P(O_{i}^{c})$ is the probability of the complement $\left(P\left(O_{i}^{c}\right)=1-P\left(O_{i}\right)\right)$. In light of the simplified network scheme presented in Figure 2b, since for all i∈{1,…,N} one has $P\left(O_{i}\right)=p_{i}P_{S}^{\left(N-i\right)}$, it follows that $P_{S}^{\left(N\right)}$ can be iteratively computed by:

(4) $$P_{S}^{\left(N\right)}=1-\prod_{i=1}^{N}\left[1-p_{i}P_{S}^{\left(N-i\right)}\right],$$

with the initial condition $P_{S}^{\left(0\right)}=1$.

For instance, considering the model presented in Figure 2b, the computation of $P_{S}^{\left(3\right)}$ implies the knowledge of its predecessor terms $P_{S}^{\left(2\right)}$ and $P_{S}^{\left(1\right)}$, which can be calculated as follows

$$\begin{array}{cc} P_{S}^{\left(1\right)} & =1-\prod_{i=1}^{1}\left[1-p_{i}P_{S}^{\left(N-i\right)}\right],\\ \; & =1-\left[1-p_{1}P_{S}^{\left(0\right)}\right],\\ \; & =p_{1}.\\ P_{S}^{\left(2\right)} & =1-\prod_{i=1}^{2}\left[1-p_{i}P_{S}^{\left(2-i\right)}\right],\\ \; & =1-\left[1-p_{1}P_{S}^{\left(1\right)}\right]\times \left[1-p_{2}P_{S}^{\left(0\right)}\right],\\ \; & =1-\left[1-p_{1}\left(p_{1}\right)\right]\left(1-p_{2}\right),\\ \; & =1-\left(1-p_{1}^{2}\right)\left(1-p_{2}\right).\\ P_{S}^{\left(3\right)} & =1-\prod_{i=1}^{3}\left[1-p_{i}P_{S}^{\left(3-i\right)}\right],\\ \; & =1-\left[1-p_{1}P_{S}^{\left(2\right)}\right]\times \left[1-p_{2}P_{S}^{\left(1\right)}\right]\times \left[1-p_{3}P_{S}^{\left(0\right)}\right],\\ \; & =1-\left[1-p_{1}P_{S}^{\left(2\right)}\right]\left(1-p_{1}p_{2}\right)\left(1-p_{3}\right),\\ \; & =1-\left(1-p_{1}\left(1-\left(1-p1^{2}\right)\left(1-p_{2}\right)\right)\right)\left(1-p_{1}p_{2}\right)\left(1-p_{3}\right). \end{array}$$

### 3.2. Expected Number of Transmissions

Let $T_{N}$ be the number of transmissions in an *N*-hop opportunistic model. For i=1,…,N, let $A_{i}$ denote the event in which the *i*-th node receives the signal from the source. Additionally, let ${𝟙}_{A_{i}}$ denote the indicator function of the event $A_{i}$, that is, it assumes 1 if the event $A_{i}$ occurs and 0 otherwise. Thus,

$$\begin{array}{c} T_{N}=N+\sum_{i=1}^{N-1}{𝟙}_{A_{i}}T_{N-i}. \end{array}$$

Taking the expectation in both sides, yields

$$\begin{array}{c} E\left[T_{N}\right]=N+\sum_{i=1}^{N-1}E\left[{𝟙}_{A_{i}}T_{N-i}\right]. \end{array}$$

Besides, for each i=1,…,N−1, in light of the law of total probability, it follows that

$$\begin{array}{cc} E[{𝟙}_{A_{i}}T_{N-i}] & =E\left[{𝟙}_{A_{i}}T_{N-i}|A_{i}\right]P\left(A_{i}\right)+E\left[{𝟙}_{A_{i}}T_{N-i}|A_{i}^{c}\right]P\left(A_{i}^{c}\right),\\ \; & =p_{i}E\left[T_{N-i}\right], \end{array}$$

where the second term is canceled since the number of transmissions sent by the *i*-th node, given that it did not receive the package, is zero, that is, $E[{𝟙}_{A_{i}}T_{N-i}|A_{i}^{c}]=0$.

Therefore, the expectation of the number of transmissions in an *N*-hop opportunistic model is given by

(5) $$\begin{array}{cc} E[T_{N}] & =N+\sum_{i=1}^{N-1}p_{i}E\left[T_{N-i}\right]. \end{array}$$

It is straightforward to note that an *N*-hop opportunistic model contains N−1 opportunistic models nested with h=1,2,…,N−1 number of hops, respectively. For instance, one has from Equation (5) that

(6) $$\begin{array}{cc} E[T_{2}] & =2+p_{1}E\left[T_{1}\right]\\ E[T_{3}] & =3+p_{1}E\left[T_{2}\right]+p_{2}E\left[T_{1}\right]\\ E[T_{4}] & =4+p_{1}E\left[T_{3}\right]+p_{2}E\left[T_{2}\right]+p_{3}E\left[T_{1}\right], \end{array}$$

and so on, where $E[T_{1}]=1$ since $P(T_{1}=1)=1$. Equation (5) provides a simple and clear manner to compute $E[T_{N}]$ instead of considering all $2^{N-1}$ possible routes, reducing significantly the computational effort.

### 3.3. Expected Number of Receptions

Similar to what was done in the previous section for the expected number of transmissions, one can summarize the total number of receptions in an *N*-hop opportunistic model, denoted by $R_{N}$, by considering two parts: the number of receptions received directly from the source and the number of receptions received from the routers. Thus, $R_{N}$ can be written as

$$\begin{array}{c} R_{N}=\sum_{i=1}^{N}{𝟙}_{A_{i}}+\sum_{i=1}^{N-1}R_{N-i}. \end{array}$$

Since ${𝟙}_{A_{i}}$ and $R_{N-i}$ are independent random variables, $E\left[{𝟙}_{A_{i}}R_{N-i}\right]=E\left[{𝟙}_{A_{i}}\right]E\left[R_{N-i}\right]=p_{i}E\left[R_{N-i}\right]$. Hence, for each i=1,…,N−1,

(7) $$E\left[R_{N}\right]=\sum_{i=1}^{N}p_{i}+\sum_{i=1}^{N-1}p_{i}E\left[R_{N-i}\right],$$

(8) $$E\left[R_{N}\right]=p_{N}+\sum_{i=1}^{N-1}p_{i}\left[1+E[R_{N-i}]\right],$$

From (8), one can see that

$$\begin{array}{cc} E[R_{2}] & =p_{1}+p_{2}+p_{1}E\left[R_{1}\right]\\ E[R_{3}] & =p_{1}+p_{2}+p_{3}+p_{1}E\left[R_{2}\right]+p_{2}E\left[R_{1}\right]\\ E[R_{4}] & =p_{1}+p_{2}+p_{3}+p_{4}+p_{1}E\left[R_{3}\right]+p_{2}E\left[R_{2}\right]+p_{3}E\left[R_{1}\right], \end{array}$$

and so on, where $E\left[R_{1}\right]=p_{1}$ as boundary. From Equation (7) one can say that the first term represents the expected number of receptions sent by the initial node and the second term computes the expected number of receptions within the nested sub-models. Equation  (8) is expressed in a computationally efficient and friendly manner to obtain $E[R_{N}]$.

### 3.4. Expected Number of Broadcast Transmissions

Let $T_{N}^{B}$ be the number of broadcast transmissions in an *N*-hop opportunistic model. It is possible to derive a scheme analogous to Equation (6) to compute $T_{N}^{B}$ as follows

(9) $$\begin{array}{cc} E[T_{2}^{B}] & =1+p_{1}E\left[T_{1}^{B}\right]\\ E[T_{3}^{B}] & =1+p_{1}E\left[T_{2}^{B}\right]+p_{2}E\left[T_{1}^{B}\right]\\ E[T_{4}^{B}] & =1+p_{1}E\left[T_{3}^{B}\right]+p_{2}E\left[T_{2}^{B}\right]+p_{3}E\left[T_{1}^{B}\right], \end{array}$$

and so on, where $E[T_{1}^{B}]=1$. Finally,

(10) $$\begin{array}{cc} E[T_{N}^{B}] & =1+\sum_{i=1}^{N-1}p_{i}E\left[T_{N-i}^{B}\right]. \end{array}$$

Note the similarity between (5) and (10) since a single initial broadcast transmission for an *N*-hop opportunist model involves *N* transmissions.

## 4. Opportunistic Model Considering Random Probabilities

Consider an opportunistic network problem with random probabilities, i.e., each link probability $p_{i}$ follows a probability density function (*pdf*) with support on (0,1). Hereinafter, those probabilities are referred to as prior distributions since they can be chosen in such a way that some prior information about them can be introduced in the model.

Let $p=[p_{1},\ldots ,p_{N}]$ be a vector with random entries following a distribution $f_{p}\left(\theta \right)$, namely $p∼f_{p}\left(\theta \right)$, where “∼” denotes “is distributed as”, θ represents a suitable vector of parameters, and $f_{p}\left(\theta \right)$ is the prior distribution. On the other hand, let *X* be a random variable of interest that depends on the vector of probabilities p. As a consequence, the *pdf* of *X* also depends on p, and one can say that *X* follows a distribution g(·) conditioned on the probabilities p, represented by X∼g(x|p). For instance, one can consider *X* as $T_{N}$, $R_{N}$ or $T_{N}^{B}$. By the law of total expectation [34], one has that the expectation of *X* can be computed as $E\left[X\right]=E_{p}\left[E\left[X|p\right]\right]$, that is

(11) $$\begin{array}{cc} E[X] & =\int_{{\left[0,1\right]}^{N}}E\left[X|p\right]\;f_{p}\left(\theta \right)dp, \end{array}$$

where E[X] is computed using two embedded expectations (a double integral), with E[X|p] referred to as the inner expectation and $E_{p}\left[\cdot \right]$ as the outer one. Note that, the subscript in the expectation represents the variable of integration, which is omitted whenever it can be easily inferred from the context.

The inner expectation E[X|p] is computed with respect to the random variable *X*, given a fixed probability vector p. This yields a function depending on p, that finally becomes a number (E[X]) when one takes the outer expectation over p. Even though (11) may appear complex, it has a simple explanation. Considering *X* as the expected number of transmissions $T_{N}$, Equation (11) states that $E[T_{N}]$ can be computed as the mean of the expected values of the number of transmissions $E[T_{N}|p]$ for all possible values of p. Since p could take infinite values, the expectation is computed integrating over the hypercube ${\left[0,1\right]}^{N}$. Thus, for a random vector of probabilities $p=[p_{1},\ldots ,p_{N}]$, one has that $E\left[T_{N}\right]=E_{p}\left[E\left[T_{N}|p\right]\right]$, and then Equation (5) can be rewritten as

(12) $$\begin{array}{cc} E[T_{N}] & =E_{p}\left[\left.N+\sum_{i=1}^{N-1}p_{i}E\left[T_{N-i}\right]\right|p\right],\\ \; & =E_{p}\left[\left.N\right|p\right]+\sum_{i=1}^{N-1}E_{p}\left[\left.p_{i}E\left[T_{N-i}\right]\right|p\right],\\ \; & =N+\sum_{i=1}^{N-1}E_{p}\left[p_{i}\right]\;E_{p}\left[\left.E[T_{N-i}]\right|p\right],\\ \; & =N+\sum_{i=1}^{N-1}E_{p}\left[p_{i}\right]\;E\left[T_{N-i}\right]. \end{array}$$

As can be seen, the only difference between Expressions (5) and (12) lies in p, where for the random probabilities case, the expected probabilities $E_{p}\left[p\right]$ are incorporated in the formula instead of the point probabilities p. For $P_{S}^{\left(N\right)}$, $E[R_{N}]$ and $E[T_{N}^{B}]$, expressions similar to those presented in (4), (7) and (10) can be written as

(13) $$\begin{array}{cc} P_{S}^{\left(N\right)} & =1-\prod_{i=1}^{N}\left[1-E\left[p_{i}\right]\;P_{S}^{\left(N-1\right)}\right], \end{array}$$

(14) $$\begin{array}{cc} E[R_{N}] & =\sum_{i=1}^{N}E[p_{i}]+\sum_{i=1}^{N-1}E\left[p_{i}\right]\;E\left[R_{N-i}\right], \end{array}$$

(15) $$\begin{array}{cc} E[T_{N}^{B}] & =1+\sum_{i=1}^{N-1}E\left[p_{i}\right]\;E\left[T_{N-i}^{B}\right]. \end{array}$$

For the probability of successful transmissions $P_{S}^{\left(N\right)}$, it has been assumed that probabilities $p_{i}$’s are independent, i.e., $f_{p}\left(\theta \right)=\prod_{i=1}^{N}f_{p_{i}}\left(\theta_{i}\right)$, where each probability $p_{i}$ follows a *pdf* $f_{p_{i}}\left(\theta_{i}\right)$, that is, $p_{i}∼f_{p_{i}}\left(\theta_{i}\right)$, and θ is a vector of parameters $\theta ={\left[\theta_{1},\ldots ,\theta_{N}\right]}^{\prime }$. The proof of (13) is beyond the scope of the present paper and hence is omitted. Expressions (14) and (15) can be easily found following the same steps in (12).

As noted, the probability of successful transmissions $P_{S}^{\left(N\right)}$, and the expectations $E[T_{N}]$, $E[R_{N}]$ and $E[T_{N}^{B}]$, only depend on the expectation E[p], regardless of the *pdf* of p. Consequently, it is possible to conclude that, for the measures of interest, to consider an opportunistic model with random probabilities $p^{*}$ is equivalent to consider a model with fixed probabilities, set as its expectations, that is, $p=E[p^{*}]$.

## 5. Comparison with Existing Methods in the Literature

There are some interesting works in the literature regarding OR models. For instance, in [25], the effect of the number of re-transmissions in each node for the case where the transmitted packet does not reach any of the candidate nodes is investigated. To this end, the authors established an analogy between a discrete-time Markov chain and OR. The key feature is that the technique can be applied to any kind of network topology, but only one possible candidate forwards the packet at each transmission, i.e., it is assumed perfect coordination among the candidates. Moreover, the destination may not be reachable from the source in just one step. In [35], a general framework for OR is proposed in terms of a probabilistic graph where each link is marked with a number between 0 and 1, representing the delivery ratio. Similarly to [25], it can be applied to any kind of network topology. Besides, a priority function is considered, establishing the priority (order relationship) among the nodes, which is needed to decide which node (among all that received the packet) will broadcast next. The main restriction is that a packet cannot be forwarded by a node with a certain priority toward nodes exhibiting lower priority.

The model proposed in this paper differs from the ones given in [25,35], essentially because all the nodes (candidates) are allowed to forward the packet toward the destination, once they receive it, leading to a higher delivery probability. Using a recursive approach, it is proposed simple closed-form expressions circumventing cumbersome mathematical expressions like the ones found in other works. In addition, to compute the full routing probability distribution, any restriction about the probability can be imposed. Even more, unlike the other two models, the model proposed here contemplates uncertainty by considering random probabilities following any unknown distribution function, for instance, uniformly distributed on an interval or following a complex bimodal distribution.

As discussed at the end of Section 4, when one considers random probabilities in the proposed model, it is sufficient to know the expected value of each probability $p_{i}$ and not its probability distribution. This feature is very advantageous since the expectations can be easily estimated, if necessary. To the best of the authors’ knowledge, this is the only work in the OR literature that can be easily replicated by the interested reader because the entire formulation has been implemented and made available through functions in a free library (see Appendix A section).

## 6. Validation of the Model

To validate the proposed model, two different 5-hop opportunistic models are investigated. The first one is a traditional OR model, considering precisely known probabilities, while the second one introduces uncertainties when considering random probabilities on a bounded interval. Simulations using $10^{6}$ Monte Carlo (MC) realizations were performed to compare the measures of interest with the ones obtained using the proposed expressions.

All methods proposed in this paper as well as numerical routines can be easily reproduced using the R software library “Opportunistic”. This library is freely available to practitioners, and it also offers a user-friendly manual (Manual available on 30 October 2021 https://CRAN.R-project.org/package=Opportunistic).

### 6.1. Precisely Known Probabilities on an OR Model

Consider a 5-hop opportunistic model with decreasing probabilities $p={\left[0.85,0.72,0.50,0.28,0.05\right]}^{\prime }$. According to Equation (2), there are seven possible routes, and their associated probabilities (mathematical expressions and values) can be found in the Appendix A.

Table 1 shows the true values of the probability of successful transmissions $P_{s}^{\left(N\right)}$, as well as the expected number of transmissions $T_{N}$, receptions $R_{N}$ and broadcasts $T_{N}^{B}$, obtained using the expressions given in Section 3, not only for the sixth node (N=5, sink), but also for all intermediate nodes. For instance, in the third column (hop 3), the value 0.9581 is the probability that the packet reaches the fourth node successfully. These intermediary values are obtained with no additional computational effort due to the recursive scheme of the model. Finally, the last column contains the MC estimates (arithmetic mean) of the interest measures for the full system. Comparing actual and estimated values (both in bold), it is possible to verify the accuracy of the proposed method.

### 6.2. Random Probabilities on an OR Model

**Figure 4:**
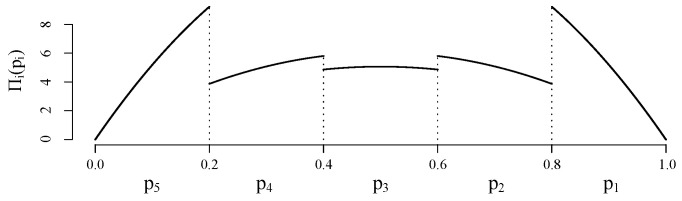
Prior distributions for probabilities $p_{i}$’s for a 5-hop opportunistic model considered for a MC simulation study.

Consider a 5-hop opportunistic model where each node forwards a packet according to a set of random probability $p^{*}$. Each probability has a *pdf* as illustrated in Figure 4. In particular, each $p_{i}^{*}$ is set to follow a doubly-truncated Beta distribution Beta(α,β,a,b) with parameters α=β=2 and truncated support on (a,b)=(1−0.2i,1.2−0.2i) for i=1,…,5. This is a convenient distribution since its support lies within the interval [0,1] and it can be limited to an arbitrary bound (a,b)∈[0,1]. For instance, $p_{2}^{*}∼\mathrm{Beta}\left(2,2,0.6,0.8\right)$.

Following the proposed model, one considers a 5-hop opportunistic model with known fixed probabilities given by $p={\left[E\left[p_{1}\right],E\left[p_{2}\right],E\left[p_{3}\right],E\left[p_{4}\right],E\left[p_{5}\right]\right]}^{\prime }$. Using some results from the literature, one has that these probabilities are given by $p={\left[0.831,0.713,0.500,0.287,0.099\right]}^{\prime }$.

For each MC realization, the probabilities are generated according to their distribution functions, and the OR process is simulated. Finally, the arithmetic means of the interest measures and the MC estimates are stored.

**Figure 5:**
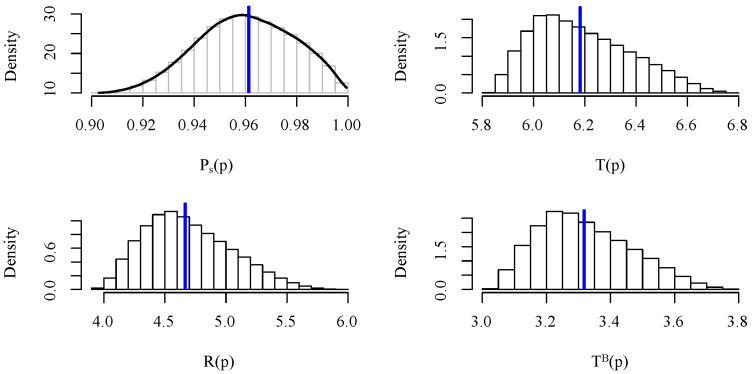
Estimated $P_{s}^{\left(N\right)}$ density and histograms for $P_{s}^{\left(N\right)}$, $T_{N}$, $R_{N}$ and $T_{N}^{B}$ when considering random probabilities. True mean values are represented by a blue vertical line.

For this particular example, the results are summarized in Table 2 and Figure 5. By observing Table 2, one can confirm the claim that considering the expected values as fixed probabilities leads to the same results than the estimates obtained via MC simulation, validating expressions (12), (13), (14) and (15). Figure 5 shows the estimated density (via smoothing kernel) for the probability of successful transmission $P_{s}^{\left(N\right)}$ and the histograms for the number of transmissions, receptions and broadcasts. The true mean values, obtained using the proposed expressions in Section 3, are represented by a blue vertical line. Note that the *pdf* of $P_{s}^{\left(N\right)}$ seems right-skewed while the probability mass functions (*pmf*’s) for $T_{N}$, $R_{N}$ and $T_{N}^{B}$ are left-skewed.

## 7. Energy Consumption for WSN Opportunist Network

This section presents a practical example where the proposed OR network model can be employed. The fundamental concepts for modeling the energy consumption on an OR wireless sensor network are exposed in this section. In wireless networks, usually, each network node has two operational modes: transmission and reception. Each mode, and the switching between them, has a specific energy cost associated, as can be seen in [Figure 2] [36]. Using the notation from [37], the costs are defined as: transmission cost ($E_{TX}$); reception cost ($E_{RX}$); and switching cost ($E_{SW}$).

The total energy consumption for a generic wireless network is denoted by $E_{T}$, and it can be obtained as a function of $E_{TX}$, $E_{RX}$, $E_{SW}$, assuming that the nominal state of a node is reception. Whenever a node is ready to transmit a packet, first it must switch from reception mode to transmission mode, then transmit the packet, and after it must switch back to the reception mode. In any opportunistic network with linear topology, the cost in Joules (*J*) for transmission of a single message can be computed by

(16) $$\begin{array}{c} E_{T}=\left\{\left(E_{TX}+2E_{SW}\right)T_{N}^{B}+E_{RX}N_{X}\right\}J \end{array}$$

where $T_{N}^{B}$ and $N_{X}$ correspond to the expected number of broadcast transmissions and the expected number of receptions, respectively. It is possible to generalize Equation (16) to take into account, for instance, the packet length or variable power transmission, by including the information about those parameters in the model, as presented in [37].

### Numerical Example

The energy costs ($E_{TX}$, $E_{RX}$ and $E_{SW}$) used in this example were obtained by [36] via experiments using modules IEEE 802.15.4 XBee Pro. In [36], the cost consumption is presented in terms of the current consumption in mA (reception: 52 [mA]; transmission: 57 [mA]). To compute the energy cost in Joules (useful for battery selection), it must be specified the supply voltage (3.3 V) and the time duration of transmissions and receptions (250 [μs]). The energy cost in Joules corresponds to $E_{TX}=4.702$ [μJ], $E_{RX}=4.29$ [μJ] and $E_{SW}=0$ [μJ].

The network used in this simulation is the same one employed in Section 6 to transmit a single package from the source to the sink. The energy consumption expected value for the case where the probabilities are precisely known is equal to 1.0503 [μJ], and for the case where the probabilities are uncertain (Figure 4) is equal to 0.3569 [μJ]. To obtain specific energy consumptions, such as for cluster or particular nodes of the OR network, one can follow the same steps from [37] [Appendix A].

## 8. Conclusions

This paper proposed a general theoretical stochastic model for opportunistic routing based on wireless networks. The results can be obtained for both precisely known and random probabilities. The random probabilities provide versatility, allowing us to model link or freight layer errors, suitable for modeling more realistic environments. Note that the proposed model corresponds to an idealized development of a network, similar to the content of a *supply–demand network*. The results can be easily obtained through an R package available for free, and let the practitioners either circumvent the use of simulation studies or provide theoretical reference values for future comparison.

Differently from the models available in the literature, based on Markov chains, the proposed technique is capable of obtaining the network parameters (total number of routes, expected number of networks broadcast, successful packet transmission and, network reception) recursively using a percolation stochastic model.

The major advantages of the proposed approach, when compared with other OR models from the literature, include: nodes (candidates) are allowed to forward the packet toward the destination, once they receive it, leading to a higher delivery probability; no constraint is imposed on the probabilities; uncertain and random probabilities following any unknown distribution function, for instance, uniformly distributed over an interval or following a complex bimodal distribution are contemplated in the proposed model; regardless of the knowledge of the probabilities, the method provides similar values to the ones obtained via MC simulation. Finally, it is important to mention that the stochastic model is an advantageous tool to compute the energy consumption of a wireless network beforehand, without requiring simulation or practical implementation. This task can be easily accomplished using the R package, which allows minimizing time and effort in engineering network design.

As future work, it is suggested the adaptation of non-linear restrictions for the OR model to determine the network parameters. One particular type of restriction is the ability to delete specific routes, being a useful feature to eliminate redundant transmissions, duplicated messages and/or congestion.  

## Figures and Tables

**Table 1:** Comparison of the true and estimated values for $P_{s}^{\left(N\right)}$, $E[T_{N}]$, $E[R_{N}]$ and $E[T_{N}^{B}]$.

	Hops					
	1	2	3	4	5	MC
$P_{s}^{\left(N\right)}$	0.85	0.9223	0.9581	0.9742	**0.9792**	**0.9791**
$E[T_{N}]$	1	2.85	6.1425	11.7731	**21.135**	**21.129**
$E[R_{N}]$	0.85	2.2925	4.6306	8.3616	**14.226**	**14.220**
$E[T_{N}^{B}]$	1	1.85	3.2925	5.6306	**9.3616**	**9.3582**

**Table 2:** Comparison of the true and estimated values for $P_{s}^{\left(N\right)}$, $E[T_{N}]$, $E[R_{N}]$ and $E[T_{N}^{B}]$.

	$P_{s}^{\left(N\right)}$	$E[T_{N}]$	$E[R_{N}]$	$E[T_{N}^{B}]$
True	0.9611	6.1876	4.6808	3.3193
Estimated	0.9601	6.1900	4.6894	3.3207

## Data Availability

Data in https://cran.r-project.org/web/checks/check_results_Opportunistic.html (accesseed on 30 October 2021).

## Appendix A

### Appendix A.1. Opportunistic R Package Tutorial

It is shown in this section how to access the methods proposed in this work through user-friendly functions available in a free R library. The Opportunistic package must be accessed using the R software, which can be downloaded at https://cloud.r-project.org/, or to be compiled online directly from cloud services as RStudio cloud accessed on 30 October 2021, https://rstudio.cloud/ or the well-known Google Colab accessed on 30 October 2021, https://colab.to/r, where is it necessary to create an account for the latter two. First, the Opportunistic R package must be installed. For this, one can use the following code in the R console

> install.packages(“Opportunistic”)

In R, > represents the prompt in the command line and must not be entered by the user. Then, load the package as

> library(Opportunistic)

Once loaded, one can call the three main functions: routes(p,delta), Expected(p) and MonteCarlo(p,M). To use these functions, the vector of probabilities p of length *N* must be provided. The first function returns the different possible routes, their frequency as well as their respective probabilities (mathematical expressions and values) when considering uncertain probabilities lying on the interval p ± delta. All results are based on the theory developed in Section 2.1. The last two functions return the probability of successful transmissions and the expected number of transmissions, receptions and broadcasts. The first function (routes), provides these measures for all nodes using expressions (4), (5), (7) and (10). The second one provides its MC estimates for the last node only. The latter also provides a progress bar so the user can estimate the processing time. By default, delta is considered to be zero (no uncertainty) and $M=10^{5}$ when the number of Monte Carlo realizations is not declared.

The results for the three main functions are shown for the same 5-hop opportunistic model with decreasing probabilities $p={\left[0.85,0.72,0.50,0.28,0.05\right]}^{\prime }$ proposed in Section 6.1. The number the possible routes is obtained by

> routes(p,delta = 0.05)				
	Freq Probability p - delta p + delta			
route 1	1	p1^5	0.32768	0.59049
route 2	4	p1^3*p2	0.34304	0.56133
route 3	3	p1*p2^2	0.35912	0.53361
route 4	3	p1^2*p3	0.288	0.4455
route 5	2	p2*p3	0.3015	0.4235
route 6	2	p1*p4	0.184	0.297
route 7	1	p5	0	0.1
Total	16}			
> True = Expected(p)				
########################################### Opportunistic Model - Theoretical results ###########################################				

Number of Hops N:	5				
Probabilities:	0.85,	0.72,	0.5,	0.28,	0.05

	1	2	3	4	5
Success Probability	0.85	0.922	0.958	0.974	0.979
Exp Transmissions	1.00	2.850	6.143	11.773	21.135
Exp Receptions	0.85	2.293	4.631	8.362	14.226
Exp Broadcast	1.00	1.850	3.293	5.631	9.362
> Estimated = MonteCarlo(p,M=10^6) ########################################### Opportunistic Model - Monte Carlo results ###########################################					
Number of Hops N:	5				
Probabilities:	0.85,	0.72,	0.5,	0.28,	0.05
Simulations M:	1e+06				
|===========================================| 100%					
Monte Carlo					
Probability System		0.979			
Exp Transmissions		21.129			
Exp Receptions		14.220			
Exp Broadcast		9.358			
